# Supplementary material for: Functional Annotations of Paralogs: A Blessing and a Curse
Source: Life (Basel). 2016 Sep 8;6(3):39. doi: 10.3390/life6030039 (PMC5041015; doi:10.3390/life6030039)
Supplement: Supplementary file 1 [file life-06-00039-s001.zip › Document S1-final.pdf]

Supplementary Materials: Functional Annotations of Paralogs: A Blessing and a Curse

Rémi Zallot, Katherine J. Harrison, Bryan Kolaczkowski and Valérie de Crécy-Lagard

Sequences selected form pubSEED to generate and compare logos

>fig|1007123.3.peg.641 Q6-carboxytetrahydropterin synthase (EC 4.1.2.50) @ Queuosine biosynthesis QueD, PTPS-I [SAR406 cluster bacterium SCGC AAA160-I06]
MFQLTRKYEFHAARKLTMLDVDHPCSQLHGHTFSVVLELSGEIDSSKGWVVDFYDIDKIYKDEIH
ALLDHKHLNDIEELSNPTTELIAKWIWNRIQPNLSGLTGVTVSEGPSYSCTYRGV

>fig|102127.3.peg.996 6-pyruvoyl tetrahydrobiopterin synthase (EC 4.2.3.12) [Geitlerinema sp. PCC 7105]>fig|102127.3.peg.996 6-pyruvoyl tetrahydrobiopterin synthase (EC 4.2.3.12) [Geitlerinema sp. PCC 7105]
MQASLTVSTHFSAAHRLARPDLDYDTNCGIYGKCARPHGHGHDYHLDVTVRGTIDPRTGMVVE
LGALHGVLQEIVIEPLDHRFLNKDIPYFADRVPTAEHIAIYIRDILQEPLRQLGVTLEKVKLYESPN
NSCEVYGTRSSNSATPATTAKPKLVLV

>fig|1075091.3.peg.1875 6-carboxytetrahydropterin synthase (EC 4.1.2.50) @ Queuosine biosynthesis QueD, PTPS-I / Folate biosynthesis protein PTPS-III, catalyzes a reaction that bypasses dihydroneopterin aldolase (FolB) [Clostridium sporogenes PA 3679]
MILIKKFKFDAAHNLIHYHGKCERLHGHTYGVVIKISGEPDKEDMVIDFTELKAIVKENVLDILDH
AYINEIIKQPTAENIAVWIWDKLYTKLKRDNCSLYEIEVWETETSGVVYSG

>fig|1075091.3.peg.976 6-carboxytetrahydropterin synthase (EC 4.1.2.50) @ Queuosine biosynthesis QueD, PTPS-I [Clostridium sporogenes PA 3679]
MYTLKVEHSFDSAHFLANYEGKCGNIHGHRWKVEIQIKSESLVQGGQLDGMVIDFGDLKKDVKS
MVDYYDHALIIEKETMREQSLSSLKEDGFRIIEVNFRPTAENFAAFFYKFMKDKGYNVKSVTVYET
PNNSATYEESGVI

>fig|1079986.3.peg.3739 6-carboxytetrahydropterin synthase (EC 4.1.2.50) @ Queuosine biosynthesis QueD, PTPS-I [Streptomyces chartreusis NRRL 12338]
MEIFREFTFEAAHRLPRVPEGHKCARLHGHSYKVTVHVEAPVEPEAGWVMDFGDIKQAFKPIDA
QLDHYYLNDIEGLDNPTSENLARWIWDRMVTELPGLSAITVRETCTSGCTYRGE

>fig|1121904.3.peg.2753 6-pyruvoyl tetrahydrobiopterin synthase (EC 4.2.3.12) [Flexithrix dorotheae DSM 6795]>fig|1121904.3.peg.2753 6-pyruvoyl tetrahydrobiopterin synthase (EC 4.2.3.12) [Flexithrix dorotheae DSM 6795]
MHNPEWSEQKNAEVFGLCNNPNYHGHNYDLIVKVTGEVDPETGYVIDLKILKSIIKDEILDKFDH
KNLNLDTPEFKSLNPTAENIAVVIWEKLRKKLEPKFDLKVTLYETERNFVEYPAS

>fig|1122169.3.peg.1233 Folate biosynthesis protein PTPS-III, catalyzes a reaction that bypasses dihydroneopterin aldolase (FolB) [Legionella shakespearei DSM 23087]
MKRYLTTVELQKESMKFSAGHTTIFSATEREPLHGHMYCVYLALTTWVEENGMTFDYRYYKERI
HVLCHQLNQTFLMPQFSPFLQYAEDDEYYYFTFNHKKIPFLKEDVTLMPLSNITVEELSRWFVNE
LIKDTEELDRHRIEKVVVKVFSAPGQSANHEWHRVK

>fig|1144275.3.peg.1442 Folate biosynthesis protein PTPS-III, catalyzes a reaction that bypasses dihydroneopterin aldolase (FolB) [Corallococcus coralloides DSM 2259]
MAPRTTTLELHKEEMKFSAGHFTIFSATHRENLHGHNFSVYVALTGEVSDDGLLSDYGPLKQAII
QRCKAWNETFFLPAHSKHLRLERDEKGNHVAHFNGEELRFLARDVTVLPVANVSLEELARVFGE
ELVGDGSAMARDHITGLVVKCASGPGQWASWEWKRDV

>fig|1178537.3.peg.3009 6-pyruvoyl tetrahydrobiopterin synthase (EC 4.2.3.12) [Bacillus sp. HYC-10]>fig|1178537.3.peg.3009 6-pyruvoyl tetrahydrobiopterin synthase (EC 4.2.3.12) [Bacillus sp. HYC-10]
MLYLSRKMYFSSLHSYRVKEWSDEYNQQVFGPCSNPNGHGHDYTLEVMVKGKLNRQSGIVVNI
TDIDKVVKSFVAENLDGKFLNMENDYFKHHIPTTENIATYLWESLDGKIDHCQIHKIRLHENNFL
YSEKEDGQLVRLTRKYHFCTAHRLHSEQLSEEENQELFGKCNNPYGHGHNYYLEVTVNGEPDPV
TGMIANLSDIDSVVEKEIMERFDHKHLNLDTEEFKDLNPTSENVAVVIWDLLSPQLTNLFKIGLYE
TEKNYFEYCGPQEDMKYGA

>fig|1178537.3.peg.3654 6-carboxytetrahydropterin synthase (EC 4.1.2.50) @ Queuosine biosynthesis QueD, PTPS-I [Bacillus sp. HYC-10]
MLSQIYPQTDHPFSFELNKDMHLSAAHFVPREEAGACSRVHGHTYTINITIAGDDLDESGFLVNF
STLKKLIHGQYDHTLLNDHEEFSSNDPYAMPTTEVVAKTVYDKVAAYLTTLANKPVCVQVFVRE
TPTSYCIYRPKRVELK

>fig|1193806.4.peg.1496 Queuosine biosynthesis QueD, PTPS-I / Folate biosynthesis protein PTPS-III, catalyzes a reaction that bypasses dihydroneopterin aldolase (FolB) [Dehalococcoides mccartyi BTF08]
MYYLSIKKHFEAAHFLRGYKGKCENLHGHRYEVALKIKTGELDECGMGADFSLLKTRLNEVLQS
YDHTCLNDLTPFDNINPSAENIAKDIYDKLKTVINKKGVELWGVEVWESPDSSVLYQPD

>fig|1237500.3.peg.5547 6-carboxytetrahydropterin synthase (EC 4.1.2.50) @ Queuosine biosynthesis QueD, PTPS-I [Nocardiopsis ganjiahuensis DSM 45031]
MRHNFETAHRLPHLGGKCTNLHGHSWWVEVSAVAPTLDSGTVVEFGTFKSALRSWIDTYLDHG
AMLGFDDPMAKLLADHGSKLFRFGAPDPLPAEEPAGDLPHPTVEAVAVLLGRVAEEALSGLAH
VPGARIDTVSVTETHVNNAVWRNGPR

>fig|1297570.3.peg.3873 6-carboxytetrahydropterin synthase (EC 4.1.2.50) @ Queuosine biosynthesis QueD, PTPS-I [Mesorhizobium sp. STM 4661]
MFRITKEFHFSASHQLTSLPPDHQCARLHGHNYIVVVELSGGELDEHGFVRDYQDLASLKHYIDG
TFDHRHLNDVLGHDRVTAECLARHFYDWCKVRLPQTSAVRVSETPKTWAEFRP

>fig|1303518.3.peg.92 6-pyruvoyl tetrahydrobiopterin synthase (EC 4.2.3.12) [Chthonomonas calidirosea T49]>fig|1303518.3.peg.92 6-pyruvoyl tetrahydrobiopterin synthase (EC 4.2.3.12) [Chthonomonas calidirosea T49]
MLIMTRRLYFAAAHRDIQGSELTGHTYTLDVAVSGHINERTGLLVNIKELDRIVKEHVLNQIDGK
CLNDRLSFFQAHLATPENIATYIAGTVHPFLPSDIHLHSVRLQPTPVLYTEWLSSLKEVSHMLLTKI
YEFSASHRLHSDSLSAEENRTLFGKCNNPNGHGHNYELEVTICGPINPITGRVLPPERLDAVVERE
VLSRYDHRYLNLDVPEFAHVVPSAEMIVKTIWERLRPCIPAPARLYRLRLYETPRNLFEYSEEDDK
EQR

>fig|1308866.3.peg.1216 6-pyruvoyl tetrahydrobiopterin synthase (EC 4.2.3.12) [Gracilibacillus halophilus YIM-C55.5]>fig|1308866.3.peg.1216 6-pyruvoyl tetrahydrobiopterin synthase (EC 4.2.3.12) [Gracilibacillus halophilus YIM-C55.5]
MLYLSRRIDFSAMHHYRIDAWTEEQNRRIFGLCSNPNGHGHDYQLEVMVRGQLNPDYGVVVNT
TDIKASVGEYVREELDGKYLNKEHPYFMKHVPTTEQLVTFLWQGIEPKLTNCELYRLRLHENPYI
VAEKGDEHMVRLTRKYHFSAAHRLHSEQLSEQENQYFFGKCNNPYGHGHNYYLEVTVCGEPDP
ITGMITDLAELDATVDQVILEKMDHKHLNLDVAEFRDVNPTSEVVAKVIYDMLSPYISNLDKVGL
WETEKNYFEYAGEEVDV

>fig|224911.5.peg.2435 Folate biosynthesis protein PTPS-III, catalyzes a reaction that bypasses dihydroneopterin aldolase (FolB) [Bradyrhizobium japonicum USDA 110]
MWELTKSFRFEAAHSLSGTTFGAASEEIHGHSFRAEVTVRGTPDPETGMVVDLGLLQRAIEDVRL
TLDHKFLNKIEALGKPTLENLSRFVWERLQHIGKLTRVSIHRDSCNESCTYYGPQG

>fig|235909.3.peg.1842 6-carboxytetrahydropterin synthase (EC 4.1.2.50) @ Queuosine biosynthesis QueD, PTPS-I [Geobacillus kaustophilus HTA426]
MMHQLYPQVAHHYRYELNKDMHIAAAHFIPHEAAGSCANVHGHTYIVNVTVAGDELDESGFL
VNFQTLKQLVHRKLDHTLLNDHSDWFDGHDPNRFPTSEVVARTIYETIQRYLDTLPHKPKCLQV
FVRETPTSYVVYRPKAGER

>fig|235909.3.peg.319 6-pyruvoyl tetrahydrobiopterin synthase (EC 4.2.3.12) [Geobacillus kaustophilus HTA426]>fig|235909.3.peg.319 6-pyruvoyl tetrahydrobiopterin synthase (EC 4.2.3.12) [Geobacillus kaustophilus HTA426]
MTRRYYFSSAHRLHSDQLTNEENQRLFGKCNNRYGHGHNYCLEVTVIGKPDPITGMVVNLAEL
DEIVNREVLVKFDHKHLNLDTDEFKQINPTAENIVIVIWELLAPHLSSLYKIGLWETQKNYFEYFG
PHKEK

>fig|272844.1.peg.1490 Folate biosynthesis protein PTPS-III, catalyzes a reaction that bypasses dihydroneopterin aldolase (FolB) [Pyrococcus abyssi GE5]
MRARIIVRTSFDAAHAVKIGDDWESLHGHTFFLEVAVEGEIKRGYIMDFTELRKIVDDIVKELNHR
NLNKIFDNPTTENVALWIAEKVEEKLPHKVKLKRVVLWEGKDNGVELEW

>fig|309798.4.peg.91 6-carboxytetrahydropterin synthase (EC 4.1.2.50) @ Queuosine biosynthesis QueD, PTPS-I / Folate biosynthesis protein PTPS-III, catalyzes a reaction that bypasses dihydroneopterin aldolase (FolB) [Coprothermobacter proteolyticus DSM 5265]
MNVCVTKAFTFDAAHNLTNYKGKCEALHGHTYRLEVTVCGTPNELENGLLMDFGDLKDLVNK
EVLSKLDHSYLNDHFEQPSTELVAMWVFETLKPHVEKLGLVLTSVKLFETATSWVEIKADASRGD

>fig|313612.3.peg.4950 6-carboxytetrahydropterin synthase (EC 4.1.2.50) @ Queuosine biosynthesis QueD, PTPS-I [Lyngbya sp. PCC 8106]
MTSNQEEIPKIKRAESNSETWMIGKEFRFEASHQLPNHDGKCARLHGHSWRGVIYVSGNKLIDA
GAKQGMIMDYEDIKKYLKPLLDDYLDHYHLNETTGLNNPTSEAIAKWIYEQLEDKIPGLVAVRID
ETCTSQCVYSKGTSHVFLL

>fig|316274.3.peg.132 6-pyruvoyl tetrahydrobiopterin synthase (EC 4.2.3.12) [Herpetosiphon aurantiacus ATCC 23779]>fig|316274.3.peg.132 6-pyruvoyl tetrahydrobiopterin synthase (EC 4.2.3.12) [Herpetosiphon aurantiacus ATCC 23779]
MPMDVFATRRFNFSASHRYWREEWSLEQNEAVFGKCTNRYGHGHNYELFVTVAGAVDPITGM
VMNMVELKRLVTMVLDQFDHKHLNEDTPYFREVIPTTENLVRVLWGLIEPQLPKGVRLAKLRLY
ENSDLYAEYFGRQQQATFNRRYEFSAAHRLHAQSLTDEANREIYGKCNNPNGHGHNYQLEVTV
DGLIDAQTGMVIDLVDMDRRVQSVLDNWDHRHLDYQVAEFAEQPSTAENIVVVLWQRLEALF
GSQLKHLRLWETANNVFDYPLSQH

>fig|324925.4.peg.1971 6-pyruvoyl tetrahydrobiopterin synthase (EC 4.2.3.12) [Pelodictyon phaeoclathratiforme BU-1]>fig|324925.4.peg.1971 6-pyruvoyl tetrahydrobiopterin synthase (EC 4.2.3.12) [Pelodictyon phaeoclathratiforme BU-1]
MNDLLRKPRKVYVSRKIEFNAAHRLFNPLFTEQENLELYGKCSNTFGHGHNYLLDVTISGIANQE
TGFLFDLKELKIILEEEIIARFDHKHLNHDVPELQECVPTTEVLAVLIWDILEKRLHNINNRELSLH
EVTIYETGKNAVRYLGE

>fig|326427.3.peg.18 6-pyruvoyl tetrahydrobiopterin synthase (EC 4.2.3.12) [Chloroflexus aggregans DSM 9485]>fig|326427.3.peg.18 6-pyruvoyl tetrahydrobiopterin synthase (EC 4.2.3.12) [Chloroflexus aggregans DSM 9485]
MVVATRRFEFSAAHRYWRDDWSAAENERVFGPYTSPYGHGHNYTLDVSITGELDERTGMVMN
MTELKAIVNEVLEEFDHKHLNLDTPYFRDQIPTTENFVRVLWRLIAARIPAHARLAHLRLYEQPD
LWVDYDGVGETQFARLYTFAAAHRLHAPALSDEENLAIYGKCNNPNGHGHNYTLEVTVQGEID
PATGMLVDLAWLDQTVHSVIDLLHLHHLDREIPAFAERPSTAENIIVYLWNELAPKLKGRLALLR
LWETRKNIFSYAGPASIPA

>fig|411474.6.peg.495 6-carboxytetrahydropterin synthase (EC 4.1.2.50) @ Queuosine biosynthesis QueD, PTPS-I [Coprococcus eutactus ATCC 27759]
MYKLKTKASFDSAHFLKDYEGKCSNIHGHRWTVEIEVGAETLEHDTQNRGMVVDFSNLKKDLR
ELADHFDHSLIMETGSLKQATEDALLAENLRIVKVDFRPTAENFAKYIYDEMTSRGYNVIEASVYE
TPNNVASYCE

>fig|415426.7.peg.182 6-carboxytetrahydropterin synthase (EC 4.1.2.50) @ Queuosine biosynthesis QueD, PTPS-I [Hyperthermus butylicus DSM 5456]
MRAPMRHRPGSGGRPGVMFLVKACTMFSAAHRIEGHPRCGKIHGHNYRVCIVVREERPLQVDL
DALEEWLERNVFQRFDHQYLNKVLAAPDKDEKLVVTSEELAVLIADMLEQAFPGRVEYVEVCET
ENLCIEYRPPRRGGFR

>fig|420247.6.peg.1034 6-carboxytetrahydropterin synthase (EC 4.1.2.50) @ Queuosine biosynthesis QueD, PTPS-I [Methanobrevibacter smithii ATCC 35061]
MKIMINGIQSNLRFSSAHVIPGHESCGYIHGHSYFVDVEIEGERAGDFEFVVDFKDVKAYTKAICN
ELDHRLLIPVYNDLIDIKDFNKKSDSIFDLKEEKTVHFKIAGKGYSIPGEDCVFLPLPYSSAEELSKFF
AETLTKKLAEKYDNLEYVAVGVNEGIGQGAIYKKVLDD

>fig|452637.4.peg.1627 6-pyruvoyl tetrahydrobiopterin synthase (EC 4.2.3.12) [Opitutus terrae PB90-1]>fig|452637.4.peg.1627 6-pyruvoyl tetrahydrobiopterin synthase (EC 4.2.3.12) [Opitutus terrae PB90-1]
MLRGTVFITRQVHFNAAHRLYNPTKSQAWNTNQYGLCTSPNWHGHNYVLEVTVRGEPDPETG
YIVDLSELKRVLHETVVDRCDHRNLNDDVDFLRNVIPTTENLVIAFWDQIEPALPAGKLHCVRLY
ETPRNFAEYFGPDAPARA

>fig|460265.11.peg.7288 6-carboxytetrahydropterin synthase (EC 4.1.2.50) @ Queuosine biosynthesis QueD, PTPS-I [Methylobacterium nodulans ORS 2060]
MKITQAFTFEAAHWLPNVPETHRCRRMHGHSYRVELTLDGPVDPHTGWVVDFYDVEHAFAPLL
AELDHHCLNDLPGLENPTAEHIAIWIWNRARPALPALSSVKVFETPLSWAEYTGD

>fig|467200.3.peg.1884 6-carboxytetrahydropterin synthase (EC 4.1.2.50) @ Queuosine biosynthesis QueD, PTPS-I [Streptomyces griseoflavus Tu4000]
MEIFREFTFEAAHRLPRVPEGHKCARLHGHSYKVTVHVEAPVDPEAGWVMDFGDIKQAFKPIDA
QLDHFYLNDIEGLENPTSENLARWIWDRMTAELPALSAITVRETCTSGCTYRGE

>fig|484018.6.peg.2512 6-carboxytetrahydropterin synthase (EC 4.1.2.50) @ Queuosine biosynthesis QueD, PTPS-I / Folate biosynthesis protein PTPS-III, catalyzes a reaction that bypasses dihydroneopterin aldolase (FolB) [Bacteroides plebeius DSM 17135]
MYTVIKRMEISASHKLKLSYESKCENLHGHNWIITVYCQAEQLNEDGMVVDFTHIKEVVKGQLD
HKNLNDVLPCNPTAENIARWICERVPHCVKVEVCESEGNRVIYEKD

>fig|485918.5.peg.3920 6-pyruvoyl tetrahydrobiopterin synthase (EC 4.2.3.12) [Chitinophaga pinensis DSM 2588]>fig|485918.5.peg.3920 6-pyruvoyl tetrahydrobiopterin synthase (EC 4.2.3.12) [Chitinophaga pinensis DSM 2588]
MIYLTRVENFNAAHKLSNPAWSKEKNEEVFGKCANENWHGHNYELHVTVKGTPDPETGFVFN
AKTLGVLIKDFIVEKIDHRNLNMDVDFMVGKFTSAENLAIGIWDQLTPHLPEGVELHCIKLYETP
RIYVEYFGAK

>fig|536232.3.peg.1673 6-carboxytetrahydropterin synthase (EC 4.1.2.50) @ Queuosine biosynthesis QueD, PTPS-I [Clostridium botulinum A2 str. Kyoto]
MYTLKVEHNFDSAHFLAGYEGKCGNIHGHRWKVEIQVQAESLVQGGQLDGMIIDFGDLKKDVK
SMVDYYDHALIIEKGTMRGQSLSSLKEDGFRIIEVNFRPTAENFAAFFYKIMKDRGYNVKSTTVYE
TPNNSATYEESGVI

>fig|536232.3.peg.879 6-carboxytetrahydropterin synthase (EC 4.1.2.50) @ Queuosine biosynthesis QueD, PTPS-I / Folate biosynthesis protein PTPS-III, catalyzes a reaction that bypasses dihydroneopterin aldolase (FolB) [Clostridium botulinum A2 str. Kyoto]
MILIKKFKFDAAHNLIHYHGKCERLHGHTYGLVIKISGERDKEDMVIDFTELKAIVKENVLDILDH
AYINEIIKQPTAENIAVWIWNKLYTKLKRDNCSLYEIEVWETETSGVVYNG

>fig|579932.3.peg.4404 6-carboxytetrahydropterin synthase (EC 4.1.2.50) @ Queuosine biosynthesis QueD, PTPS-I [Streptomyces sp. FXJ7.023]
MEIFREFTFEAAHRLPNVPEGHKCARLHGHSYKVIVHVEAPVDPESGWVMDFGDLKKAFKPLEA
QLDHYYLNDIEGLENPTSEVLARWIWERLKPTLPDLSALTVRETCTSGCTYRGE

>fig|662475.4.peg.1371 6-carboxytetrahydropterin synthase (EC 4.1.2.50) @ Queuosine biosynthesis QueD, PTPS-I [Haloarcula californiae ATCC 33799]
MSQRLSKADNTLADAGERELVVGGDRPLRISAGHRLLHHDGKCSRPHGHNYEVTVRVTGELTD
EGWVVDKGEITDVIDEWDHRFLLEAGDPLVEAFDASGDGDAVVVLDHPPTAEVMAAILEQRLA
DRLPETVSDVAVSVRETSELCVR

>fig|665937.3.peg.3439 6-carboxytetrahydropterin synthase (EC 4.1.2.50) @ Queuosine biosynthesis QueD, PTPS-I [Anaerostipes sp. 3_2_56FAA]
MYQLNTHASFDSAHFLAGYEGKCRNLHGHRWKLSVTIQKEKVEEQGQTRGMVVDFGELKDKV
KEIADEFDHALIMEKAH

>fig|70601.1.peg.620 Folate biosynthesis protein PTPS-III, catalyzes a reaction that bypasses dihydroneopterin aldolase (FolB) [Pyrococcus horikoshii OT3]
MKSRIIVRTSFDAAHAVKVGDHWEDVHGHTFFLEVAIEGEIKNGYVMDFLELRKIVEEITKELDH
RNLNNIFENPTTENIALWIGERIRDKLPPYVKLKRVVLWEGKDNGVELEW

>fig|746697.3.peg.2173 6-pyruvoyl tetrahydrobiopterin synthase (EC 4.2.3.12) [Aequorivita sublithincola DSM 14238]>fig|746697.3.peg.2173 6-pyruvoyl tetrahydrobiopterin synthase (EC 4.2.3.12) [Aequorivita sublithincola DSM 14238]
MNKIAIVRCEHFNAAHRLQNKNWSDEKNKAIFGKCNNPNYHGHNYELEVKVIGPCDPETGYVI
DTKILSDLIKQEILDRFDHKNLNLDTLEFKDLNPTAENIAITIYNLLRPAIEAKLELKIKLYETPRNY
VEYPH

>fig|755731.4.peg.3342 6-carboxytetrahydropterin synthase (EC 4.1.2.50) @ Queuosine biosynthesis QueD, PTPS-I [Clostridium sp. BNL1100]

MEQITITKEFTWDMAHMLAGHLGLCKNLHGHTYKMQVEICNKKDSVCERQDMVMDFKDLKDI
IKEKIIDSFDHSFAYWKGSDDPVEIQIADALTKNGRRVVPLDFRPTAEKMAIYFHELIKEDFESKGF
EIKKIKLWETPTSFAEYTSEGKDD

>fig|857392.3.peg.196 6-pyruvoyl tetrahydrobiopterin synthase (EC 4.2.3.12) [Marinilabilia sp. AK2]>fig|857392.3.peg.196 6-pyruvoyl tetrahydrobiopterin synthase (EC 4.2.3.12) [Marinilabilia sp. AK2]
MKVSVYRKEHFNAAHRLHNPKWSFEKNQTVFGKCNNPNYHGHNYDLIVKLDGPIDPETGYVY
DMKVLSDLIKVHVLNKFDHKNLNIDTDEFKNLNPTAENIAVVIWNILRKEIDLKFDLTIRLYETER
NFVEYSGD

>fig|945713.3.peg.1337 6-pyruvoyl tetrahydrobiopterin synthase (EC 4.2.3.12) [Ignavibacterium album JCM 16511]>fig|945713.3.peg.1337 6-pyruvoyl tetrahydrobiopterin synthase (EC 4.2.3.12) [Ignavibacterium album JCM 16511]
MVYITRRETFAAAHRLFIPELSDEENLKIFGKCSHPNWHGHNYTLEVVIAGEINPETGFVLDIKKL
KEIIHKFVIDKVDHKNLNLDTDFMKGLIPTSENICIAIWNQLKDKIPSGKLYSVKLYETENNYFEYR
GE

>fig|945713.3.peg.354 6-carboxytetrahydropterin synthase (EC 4.1.2.50) @ Queuosine biosynthesis QueD, PTPS-I [Ignavibacterium album JCM 16511]
MKIAKAFRWEMGHRLPEHFGLCKNIHGHSYKMIVEFEGELNKDQMVIDYYDVEKIINPIINQLD
HAFMVNTNDKIVLDFLEKMNSKKVVVDFDATAENICKFLLSEISKSNLPENINSVKVRVYETQFD
YAEDTLQLK

>fig|946235.3.peg.2633 6-carboxytetrahydropterin synthase (EC 4.1.2.50) @ Queuosine biosynthesis QueD, PTPS-I [Oceanobacillus sp. Ndiop]
MYGFTIVENLQKFDKDIKRHELKYHRERVLISKEFTFDAAHHLHCYQGKCKNLHGHTYRAVFGI
SGYTDEIGILIDFSEIKSIWKEQIEIHLDHRYLNETLPKMNTTAENMVVWIYEKMNQVLINRPDDC
RLEFVKLYETPTSYAEVRREWMIHE

© 2016 by the authors; licensee MDPI, Basel, Switzerland. This article is an open access article distributed under the terms and conditions of the Creative Commons by Attribution (CC-BY) license (http://creativecommons.org/licenses/by/4.0/).
